# Imaging Spectroscopy and Machine Learning for Intelligent Determination of Potato and Sweet Potato Quality

**DOI:** 10.3390/foods10092146

**Published:** 2021-09-10

**Authors:** Wen-Hao Su, Huidan Xue

**Affiliations:** 1 Department of Agricultural Engineering, College of Engineering, China Agricultural University, Beijing 100083, China; wenhao.su@cau.edu.cn; 2 School of Economics and Management, Beijing University of Technology, Beijing 100124, China

**Keywords:** imaging spectroscopy, machine learning, food quality, potato, intelligent detection

## Abstract

Imaging spectroscopy has emerged as a reliable analytical method for effectively characterizing and quantifying quality attributes of agricultural products. By providing spectral information relevant to food quality properties, imaging spectroscopy has been demonstrated to be a potential method for rapid and non-destructive classification, authentication, and prediction of quality parameters of various categories of tubers, including potato and sweet potato. The imaging technique has demonstrated great capacities for gaining rapid information about tuber physical properties (such as texture, water binding capacity, and specific gravity), chemical components (such as protein, starch, and total anthocyanin), varietal authentication, and defect aspects. This paper emphasizes how recent developments in spectral imaging with machine learning have enhanced overall capabilities to evaluate tubers. The machine learning algorithms coupled with feature variable identification approaches have obtained acceptable results. This review briefly introduces imaging spectroscopy and machine learning, then provides examples and discussions of these techniques in tuber quality determinations, and presents the challenges and future prospects of the technology. This review will be of great significance to the study of tubers using spectral imaging technology.

## 1. Introduction

As plant foods grown worldwide, both potato and sweet potato tubers belong to the family of solanales. Such tubers play an important role in human diets and have made significant contributions to sustainable agricultural development and food supply [1,2]. Unlike meat, soybean, and dairy products [3,4,5], potatoes and sweet potatoes are important sources of carbohydrates and are rich in protein, calcium, and vitamin C. They can be used as staple foods, animal feeds, and for other purposes [6,7,8]. Sweet potato is a good source of natural health compounds such as anthocyanins and beta-carotene [9]. Potato is a high-yielding starch crop, which can produce more calories per unit area and unit time than other food crops such as wheat, maize, and rice [10]. The starch of fresh potatoes is not easy to be directly digested, thus most potatoes are processed into French fries, potato chips, and mashed and dehydrated foods [11].

The end use and the price of tubers are greatly affected by their variety and quality characteristics including physical characteristics (such as color and texture) and chemical compositions (such as protein and vitamins) [12]. High-throughput characterization of their quality parameters will facilitate the development of new tuber varieties. The rapid differentiation of tuber varieties allows them to be effectively integrated into the variety protection and genetic resource management. Growers may prefer to choose resistant varieties that grow well in adversity environments (such as drought and flood). Manufacturers need to quickly determine whether the nutritional quality of tubers meets their expectations. 

Traditional sampling detection methods based on liquid chromatography (LC), enzyme-linked immunosorbent assay (ELISA), and gas chromatography-mass spectrometry (GC-MS) are inefficient and unsuitable for detections of large sample sets [13,14,15,16,17]. Especially for the food industry, smart technology is urgently needed to automatically evaluate the quality characteristics of a huge number of agricultural products. Non-destructive techniques based on biosensors, computer vision, fluorescence imaging, and vibration spectroscopy have received increasing attention and have been used to quickly assess the quality attributes of tuber products [18,19,20,21,22,23,24,25,26,27,28,29]. Imaging spectroscopy can obtain the continuous spectral response of each point of an image in the visible (Vis) and near/mid infrared (NIR/MIR) ranges [24,30,31]. This technology can provide detailed characteristic parameters for non-destructive quality evaluation of foods [32,33,34]. 

In recent years, the combination of imaging spectroscopy and machine learning has achieved rapid monitoring of the quality attributes of different foods (including meats, grains, and fruits) [35,36,37,38,39,40,41,42]. As far as we know, the application of imaging spectroscopy in quality detections of potato and sweet potato tubers has not yet been reviewed. Therefore, this article will analyze the application of the technology in intelligent determination of potato and sweet potato tubers.

## 2. Imaging Spectroscopy and Machine Learning

Imaging spectroscopy integrates the main features of imaging and spectroscopic technologies, which can simultaneously acquire spatial and spectral information of an object [23,39,43,44,45]. This technology has been widely used in the quantitative determination and visualization of food physical and chemical values. In a hyperspectral image, each pixel contains a continuous spectrum composed of hundreds of wavebands [15,46,47]. The 3-dimension (3-D) spectral image with two spatial dimensions and one spectral dimension can be generated by area scan (tunable filter), line scan (pushbroom), or point scan (whiskbroom) [48]. As the successor of hyperspectral technology, multispectral technology can obtain several discrete spectral data from the test sample to characterize a certain characteristic parameter of the object of interest [49,50]. The Vis region (380–780 nm) contains spectral information related to color characteristics. The NIR spectrum is mainly in the range of 780–2500 nm, while the MIR spectrum is in the range of 2500–25,000 nm. The far infrared (FIR) spectrum is in the farther spectral range (25,000–300,000 nm). NIR and MIR spectra have higher energy than FIR spectra. These two spectra are more suitable for analyzing fingerprint information related to chemical components [51,52]. NIR spectrum is used to analyze the stretching and bending of chemical bonds, including O–H, S–H, N–H, and C–H [53]. MIR spectrum is mainly related to basic vibration and rotational vibration structure [54], which contains characteristic information related to chemical functional groups [55,56].

**Figure 1:**
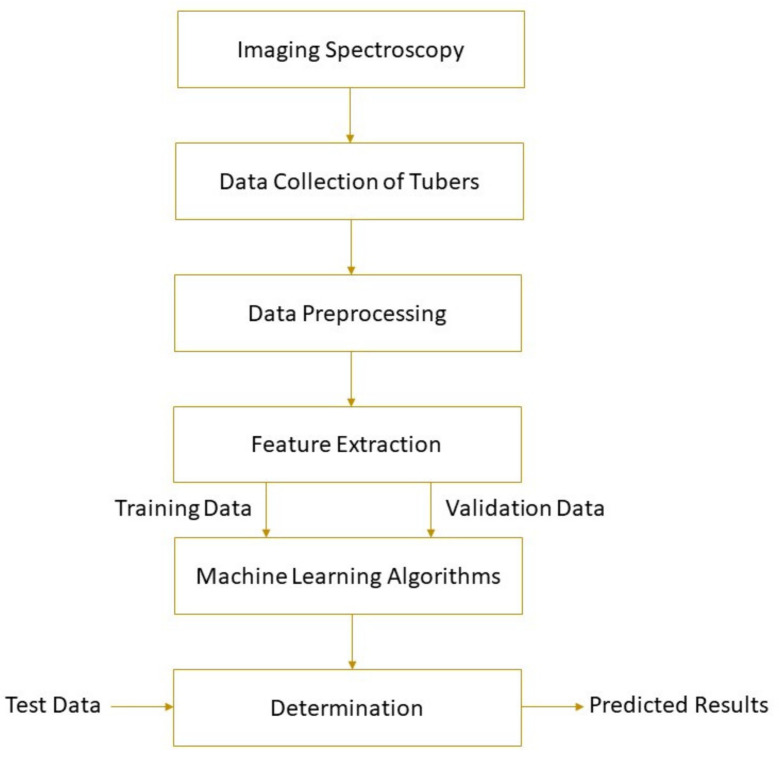
A typical schematic of imaging spectroscopy for tuber quality determinations.

The spectral parameters of the detected object and its physical or chemical properties can be correlated by machine learning. Machine learning uses mathematical algorithms to explore the rules that exist in big data to assist decision-making, involving unsupervised learning and supervised learning. More information about machine learning can be found elsewhere [57]. Based on the establishment of the calibration model, the parameter values of unknown samples can be predicted. Machine learning methods, such as principal component regression (PCR), hierarchical cluster analysis (HCA), support vector machine (SVM), partial least squares regression (PLSR), multiple linear regression (MLR), locally weighted partial least squares regression (LWPLSR), artificial neural network (ANN), and least square support vector machine (LS-SVM), have been widely used in food analysis [58,59,60,61,62]. Feature variable selection based on genetic algorithm (GA) [63], competitive adaptive reweighted sampling (CARS) [64,65], first-derivative and mean centering iteration algorithm (FMCIA) [66], regression coefficient (RC), successive projection algorithm (SPA) [67], and principal components analysis (PCA) [58] help to eliminate the feature overlap of continuous spectral information, which is conducive to the development of more robust and simplified machine learning models [68]. A high-performance model requires higher determination coefficients for cross-validation (*R*^2^_CV_) and prediction (*R*^2^_P_), correlation coefficients for prediction (*R*_P_), and lower root mean square errors for cross-validation (RMSECV) and prediction (RMSEP). Figure 1 shows the schematic of a general framework for tuber quality determination based on imaging spectroscopy. Detailed applications of the technology are given in the following section.

## 3. Applications for Tuber Quality Assessment

The concept of agricultural intelligent sensing has attracted widespread attention. In the past few years, many scientists have studied the feasibility of imaging spectroscopy in rapid quality assessments of potato and sweet potato tubers. This section provides an overview of developments and applications of this technology as listed in Table 1.

### 3.1. Physical Properties

Color is a combination of visible light reflected or emitted from an object [103]. It is the primary factor that consumers consider in determining food quality [104,105]. Foods can be quickly graded based on their color to achieve rapid food quality control. Hyperspectral technology is an effective method for tuber color analysis. Vis/NIR hyperspectral imaging was used to monitor the color of potato slices during air drying. PLSR combined with feature wavelength selection methods such as selected interval partial least squares regression (iPLSR) yielded the *R*^2^_P_ as high as 0.91 [85,91]. Then, the hyperspectral imaging was employed to measure the color index (L*, a*, b*, browning index (BI), L*/b*) of fresh-cut potato tuber slices [77]. Compared to PLSR, higher accuracies were obtained by using least squares support vector machine (LSSVM) and feature wavelength selection methods including SPA and CARS. These studies showed that Vis/IR hyperspectral technology can be used to accurately and rapidly determine the color of potatoes during processing.

The foods that consumers are willing to buy need to have acceptable sensory texture. Food texture is generally determined by the sensory performance of the structural characteristics of the product [106,107]. Traditional methods (such as texture profile analysis) of detecting the structural characteristics of foods have low efficiency [108]. Imaging spectroscopy has received greater attention in the evaluation of the texture of potato products. The textural properties of potato and sweet potato based on MIR spectra (4000–600 cm^−1^) were measured during microwave baking [55]. The authors reported that LWPLSR using feature wavelengths in the fingerprint region (1500–900 cm^−1^) presented better performances than PLSR in determination of related textural parameters including chewiness, resilience, hardness, gumminess, cohesiveness, and springiness, with the highest *R*_P_ value of 0.881. Their research concluded that the textural property of potatoes and sweet potatoes can be reliably evaluated using FT-MIR imaging spectroscopy.

**Figure 2:**
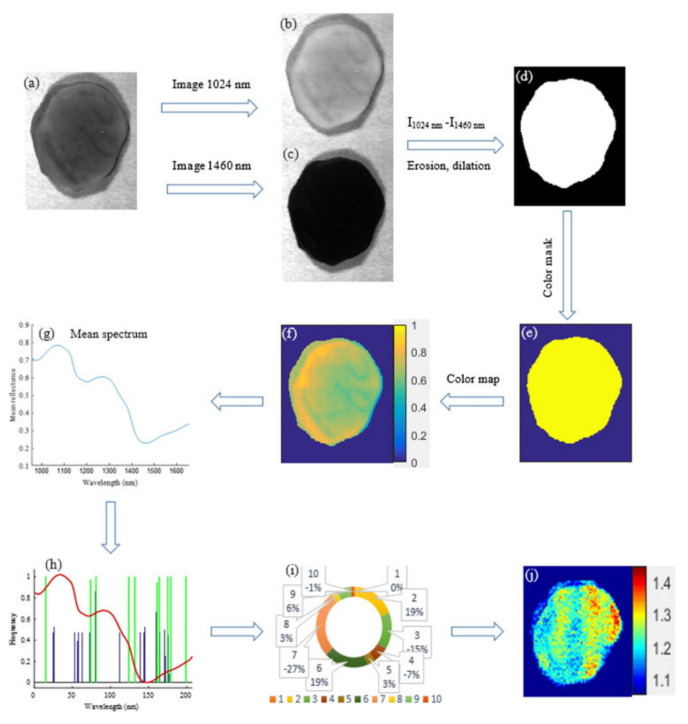
Visualization process of potato SG based on hyperspectral imaging. (**a**) The corrected hyperspectral image, (**b**) band image at 1024 nm, (**c**) band image at 1460 nm, (**d**) mask image, (**e**) color mask, (**f**) spectral reflectance index, (**g**) average spectrum, (**h**) feature variable selection, (**i**) feature variable reduction, and (**j**) distribution map of SG [93].

The quality of tubers is indirectly affected by specific gravity (SG) and water binding capacity (WBC). The SG is usually significantly affected by climate, irrigation, and soil conditions. Potatoes with a higher specific gravity can produce more products (such as potato chips and French fries) [109,110]. WBC refers to the ability of food tissue to retain water under external pressure (such as gravity, heating), which can affect the sensory quality of foods [111,112]. Foods with poor WBC are normally more easily damaged. Vis/NIR hyperspectral imaging and machine learning methods such as PLSR were used to measure the SG of sliced potato samples (Figure 2) [97]. Compared with the Vis/NIR spectra (400–1000 nm), higher accuracy was obtained by hyperspectral imaging using the NIR spectra (900–1700 nm). The locally weighted principal component regression (LWPCR) using generalized logarithm spectra (GL_S_) and power spectra (P_S_) achieved better performance than that of PLSR in determinations of SG (*R*^2^_P_ = 0.98) and WBC (*R*^2^_P_ = 0.97), respectively [93]. The models developed using feature wavelength selection methods including FMCIA-RC and GA-RC showed equivalent results to those using full-wavelength spectra.

The effectiveness of both GA-RC and FMCIA-RC was preliminarily demonstrated. The robustness of such methods should be further validated. The experimental design considerations should be more carefully considered and justified in future research. The relevance of variable selection methods should be evaluated over a longer period of time. Although good combinations of variables were selected, future work is still required. To choose the best set of feature wavelengths, the FMCIA should be properly benchmarked against the state of the art variable selection approaches such as GA [113]. The robustness of the selected wavebands could be further evaluated by other approaches such as performing the selection on perturbed datasets. This can be achieved based on performing the selection for different calibration and validation splits and evaluating if the same combination is always chosen. During real-time applications, the combination of several most important wavelengths can be separated using spectral filter arrays that are sensitive to these specific wavelengths [114]. In addition, the convergence of GA-PLS is acceptable as the convergence error of the model using several feature variables selected by GA-PLS is similar to that of full wavelength models using hundreds of wavelengths. As the majority of irrelevant variables were eliminated, the model storage capacity based on GA-PLS was reduced and the model computing speed was improved.

### 3.2. Chemical Components

Potatoes and sweet potato tubers are valuable substitutes with acceptable protein yields per hectare. Protein content is an important indicator in the selection of superior tuber genotype. The potential of spectroscopy and machine learning was investigated to determine the tuber protein. The PLSR model using NIR spectra obtained good result in the protein determination of potato and sweet potato with the highest *R*^2^_P_ of 0.98 [115,116]. The developed PLSR model showed great potential in selection of tuber cultivar with high protein content. Dry matter contains soluble and insoluble carbohydrates, which is an equivalent predictor of food flavor and consumer preference [117,118,119,120,121]. Hyperspectral imaging combined with LWPLSR, PLSR, and MLR were used for measuring the dry matter of potato and sweet potato [122]. The highly satisfactory results (*R*^2^_P_ = 0.962) were obtained using the MLR model [86]. The results showed that the models using exponent spectra (E_S_) achieved higher performance than those using other spectra including reflectance spectra (R_S_) and absorbance spectra (A_S_). Further investigation about theoretical logic of E_S_ transformation will be a direction of future research.

Starch is a glucose polymer composed of hundreds of glucose units, which can be used to assess the growth potential of crops [123]. The NIR hyperspectral technology was studied to predict the starch of sweet potato and potato [124]. The PLSR model showed acceptable accuracy (*R*^2^_P_ = 0.70) [96]. Compared with PLSR, MLR showed better performance in predicting potato and sweet potato starch, with the *R*^2^_P_ as high as 0.97 [125,126]. The FMCIA-MLR and FMCIA-PLSR developed using six feature variables obtained similar results with *R*^2^_P_ of 0.962 and 0.963 for tuber DMC and SC, respectively. Then, hyperspectral data collected from three sites of potatoes (the top, umbilicus, and middle regions) were preprocessed using standard normal variate (SNV) and three wavelength selection methods including CARS, iterative variable subset optimization (IVSO), and the variable iterative space shrinkage approach (VISSA). Then, the starch content (SC) of different potato varieties was determined based on linear PLSR and nonlinear support vector regression (SVR) models. Based on selected feature wavelengths, the CARS-SVR model achieved the highest accuracy with the *R*_P_ of 0.93 [74]. Besides the whole potato, the SC of fresh-cut potato slices was also measured. After multivariate scattering correction (MSC) preprocessing, the CARS-PLSR model exhibits the best performance with the *R*_P_ of 0.95 [75].

Soluble carbohydrates mainly involve sugars, including disaccharides (such as sucrose) and monosaccharides (such as fructose and glucose) [127,128]. Hyperspectral imaging technique was tested to determine potato glucose and sucrose. Based on the iPLSR for feature variable selection, machine learning methods including k-nearest neighbor (KNN) and PLSR models achieved better results in glucose prediction (*R*^2^_P_ = 0.88) compared with that in sucrose prediction (*R*^2^_P_ = 0.36) [129]. This imaging technique combined with SPA–SVR and CARS–MLR was capable of visualizing the spatial distribution of soluble solid content (SSC) in sliced sweet potatoes [80]. Moreover, total anthocyanin (TA) and moisture content in processed potato and sweet potatoes were detected during convective hot-air drying and microwave drying [51,94]. Results showed that hyperspectral imaging and PLSR using spectral and GLCM features was effective to monitor different levels of sweet potato moisture content and TA [78]. Based on five NIR feature wavelengths (961, 1066, 1084, 1173, and 1234 nm), PLSR obtained the highest accuracy (*R*^2^ = 0.98) for moisture content prediction in steamed and dried purple sweet potatoes [79].

The contamination of foodborne Escherichia coli on the surface of fresh-cut potatoes was detected using Vis-NIR hyperspectral imaging (400–1000 nm). The non-linear back-propagation neural network (BPNN) model coupled with GA showed higher accuracy (*R*^2^ = 0.98), in comparison with that of the linear regression models [76]. Su and Sun [92] reported that hyperspectral imaging (900–1700 nm) combined with the three-layer back propagation artificial neural network (TBPANN) model obtained higher accuracy than PLSR in detection of the volatility of tuber compositions (VTC) and prediction of the tuber cooking degree (TCD). Based on eight feature variables, the FMCIA-TBPANN model achieved good results with *R*^2^_P_ of 0.97 for VTC and 0.98 for TCD. In addition, NIR hyperspectral imaging and FT-MIR microspectroscopy were combined to determine sweet potato varieties and to measure tuber cooking loss (CL) [54]. Based on PLSDA, PLSR, and SVMR models, it was confirmed that both spectroscopic techniques provided characteristic information about tuber variety and CL.

### 3.3. Varietal Authentication

Variety identification plays a key role in breeding and production of tubers. Kasampalis, Tsouvaltzis, Ntouros, Gertsis, Moshou, and Siomos [69] explored the possibility of hyperspectral imaging coupled with PLS-based methods to classify three genotypes of potatoes into respective cultivars. The PLSR and PLSDA models combined with feature selection algorithms including variance inflation factor, variable importance scores, and GA showed similar performance to that using full-wavelength spectra, indicating the effectiveness of the feature selection techniques. To differentiate pure and adulterated sweet potato, Ding, Ni, and Kokot [21] utilized machine learning algorithms to group the NIR reflectance spectra from many different types of tuber samples. Their results showed that the purple and white sweet potato varieties could be accurately discriminated from each other as well as from different adulterated purple sweet potato samples. Furthermore, NIR hyperspectral imaging and PLSDA have been effectively utilized for potato variety classification [23,51]. The PLSDA model coupled with PCA achieved the 100% identification accuracy of sweet potato cultivars [90]. Nevertheless, the robustness of the classifiers against within class variability (such as different batches, harvesting seasons, origins, etc.) was not investigated. The classification investigated is limited to distinguishing samples from one batch for class 1 from samples from one batch from class 2. In future study, methods should be described more carefully with clear motivations for choices made. Much deeper research is recommended to confirm the robustness of classifiers. For instance, further steps including using samples from several batches and samples from different harvesting seasons would improve the robustness of the models developed.

### 3.4. Defect Aspects

The rapid detection of tuber surface defects based on high-throughput methods is helpful for the selection and breeding of disease-resistant varieties. Tuber defects are closely related to their prices and consumers’ purchasing intentions. Scab is a common skin disease that reduces the quality of potatoes. Based on the hyperspectral imaging system, the SVM classifier was used to identify common scabs on potatoes with an accuracy rate of 97.10% [100]. The hyperspectral imaging in the Vis/NIR region (400–1700 nm) achieved the rapid detection of potato defects such as scabs, surface bruises, holes, and sprouts [130,131]. Specifically, the potato sprouts were detected by supervised multiple threshold segmentation model (SMTSM) [70]. The results showed that the SMTSM coupled with Canny edge detector was effective in detection of potato germination with a high accuracy (89.85%), which demonstrated that the precision of the proposed methods. Furthermore, the Vis-NIR hyperspectral imaging showed a higher performance than other optical techniques including Vis-NIR interactance spectroscopy and NIR transmittance spectroscopy in determinations of potato sprouting activity [71]. Moreover, seven types of potato defects were classified by LS-SVM using spectral and textural features of multispectral images at 690, 757, and 927 nm, yielding the classification accuracy of 90.70% [83]. Three severity levels of bruised potatoes were successfully identified from healthy ones. Methods including Savitzky-Golay smoothing, second derivative, and optimized simulated annealing algorithm were used for data pre-processing and dimensionality reduction. Based on 12 feature wavelengths (657, 667, 678, 693, 709, 714, 750, 760, 776, 787, 808, 839 nm) selected, the SVM obtained a correct recognition rate of 100% for potatoes with minor bruise [84]. Hyperspectral imaging coupled with variable importance in projection analysis successfully detected potato tubers infested with root-knot nematodes with 100% accuracy [72]. The internal Zebra chip disease of potatoes was detected based on hyperspectral imaging (550 nm–1700 nm) [73]. The developed PLSDA model using 34 variables selected basing on the variable importance in projection (VIP) achieved an accuracy of 92% for identifications of Zebra chip infected whole potatoes. Further increase of the accuracy may be achieved using deep learning methods. In addition, potato hollow heart disease is a physiological disorder, occurring inside the tuber products. Based on the hyperspectral imaging in the reflectance mode, the SVM model was used to identify the hollow heart in potato tubers, yielding the correct classification of 89.10% [101]. The accuracy increased to 100% by hyperspectral imaging in the semi-transmission mode [132].

## 4. Challenges and Future Prospects

In general, the feasibility of imaging spectroscopy and machine learning in intelligent determination of potato and sweet potato quality has been confirmed by empirical studies. Portable spectroscopy systems allow users to get real-time evaluations of food quality parameters while reducing operational uncertainty and response time. The drawback of traditional spectroscopic methods is that spectral data are collected from a single point or from a small portion of tested samples which may not guarantee data accuracy and representativeness. The NIR point spectroscopy would provide a mean spectrum of several single points (average measurement) of a sample, irrespective of the area of the sample scanned. As the spectra collected are averaged to provide a single spectrum, the information on spatial distribution of constituents within the sample is thus lost. Hyperspectral imaging is an advanced spectroscopic technique with the advantage of acquiring spatially distributed spectral information at each pixel of an object, which is helpful to evaluate the heterogeneity of spectral signature captured from center and ends of the sample. Although values of predicted concentrations were verified and comparable to the measured values based on reference methods, to further verify these results, samples of variability including different batches, harvesting seasons, and origins should be investigated in future research.

The developed machine learning methods with effective wavelength selection showed greater ability for food quality assessment. There is no unique method to select wavelengths for a particular study. FMCIA demonstrated good performance, but further research to improve and demonstrate the robustness of the algorithm and the logic behind should be carried out in future. Additionally, future work is required to further investigate other chemometrics methods. Nonlinear modelling algorithms, such as LWPLSR and LWPCR based models, showed higher performances than linear methods. Although PLSR-based algorithms are recognized data-mining approaches, further studies are needed to improve the prediction precision and comprehensively apply them to practical uses. More studies are needed to further validate the performance of these approaches, and to develop novel simplified models in visualizing tuber quality parameters. Further study should also be conducted to monitor the change of other chemical compositions (such as ascorbic acid) in potato and sweet potato tubers. In recent years, deep learning algorithms have become increasingly popular [133]. One of the main reasons is the scalability of the data sets and the performance growth of deep learning in training phase. The availability of parallel processing and large-scale data sets simplifies the deep learning research. Deep neural networks may perform well in image classification of various foods, but they rely on a large number of labeled samples for model training [134]. Additionally, the algorithm is not sufficient enough to identify objects with high occlusion. The training data set is better to be large enough to prevent overfitting. The acquisition of large data sets often requires a large number of images to be annotated, which is a high labor cost [135].

Based on these chemical-free evaluation approaches, the sample preparation time is significantly decreased, and the errors emerged during subjective judgement are greatly reduced. On behalf of the regulatory inspection and the goal to guarantee superior product quality in food industry, imaging spectroscopy has replenished the new knowledge of determinations of food quality parameters. Given the flourishing innovation and progress in data analysis and modeling recently, it is anticipated that such imaging spectroscopy will gradually become the prevailing measurement method for quality evaluations of food products in both laboratorial and industrial scales. Thus, the applications of imaging spectroscopy have been epitomized as potential tools for quality evaluations of food products.

The depth of the analyses can be improved in future with respect to the following aspects:

(a) the robustness of the models against group variability. This can be done by leaving an entire batch or cultivar out and testing if the models still provide good predictions. Other influencing factors with different variabilities, including samples from various batches, harvesting seasons, origins, and milling processes, should be considered;
(b) the robustness of the selected set of wavebands. This can be done by performing the selection for different calibration and validation splits and evaluating if the same combination is always chosen. Additionally, different sources of samples can be used to validate the selected feature variables;
(c) carefully benchmarking the new methods against state-of-the-art ones and evaluating whether the differences in prediction performance are significant.

It has been implied that the existing spectral imaging systems are still in the developmental stage, and new strategies should be proposed to develop real-time and low-cost detection systems for food industry. With the further joint development of artificial intelligence and spectral imaging techniques, it could be anticipated that more advanced optical and imaging instruments will be established to simultaneously acquire spectral and spatial information of test specimens at laboratory and industrial scales.

## 5. Conclusions

In this review, the latest application of imaging spectroscopy in potato and sweet potato quality assessment has been confirmed. Based on the chemical-free evaluation approaches, the sample preparation time has been greatly reduced and the errors emerging during subjective judgement decreased. This imaging technology has great potential in determining the physical properties, chemical compositions, variety identification, and defects of potatoes and sweet potatoes. This technology has been added to the knowledge base for the determination of potato and sweet potato quality parameters. Given the recent boom in innovation and the advancement in data analysis and machine learning, it is expected that imaging spectroscopy will gradually become the mainstream measurement method for food quality assessments at laboratories and industrial scales. However, more work is required to successfully implement this technology in the food industry for real-time applications. Since spectral information can be explored based on machine learning to link the measured reference values, optimal prediction models could be generated to quantify the quality parameters of these foods.

## Figures and Tables

**Table 1:** Imaging spectroscopy for tuber quality assessment.

Quality Parameter	Sample Type	Spectral Region	Optimal Model	Accuracy	Reference
Freshness, Cultivar	Potato	Vis-NIR	PLSR	0.98 for freshness, 93% for cultivar discrimination	[69]
Sprout	Potato	Vis-NIR	SMTSM	89.28%	[70]
Sprouting activity	Potato	Vis-NIR	KNN, PLSDA	90%	[71]
Root-knot nematodes	Potato	Vis-NIR	PLS-SVM	100%	[72]
Zebra chip disease	Potato	Vis-NIR	PLSDA	92%	[73]
Starch	Potato	Vis-NIR	SVR	*R*_P_ = 0.93	[74]
Starch	Potato	Vis-NIR	PLSR	*R*_P_ = 0.94	[75]
*Escherichia coli*	Potato	Vis-NIR	BPNN	97.60%	[76]
Color, moisture content	Potato	Vis-NIR	LSSVM	*R*^2^_P_ = 0.84 for color, *R*^2^_P_ = 0.77 for moisture content	[77]
TA, moisture content	Sweet potato	Vis-NIR	PLSR	*R*^2^_P_ = 0.87 for TA, *R*^2^_P_ = 0.86 for moisture content	[78]
Moisture content	Sweet potato	NIR	PLSR	*R*^2^_P_ = 0.95	[79]
SSC	Sweet potato	Vis-NIR	SVR	*R*^2^_P_ = 0.86	[80]
Sulfite dioxide residue	Potato	NIR	SVM	95%	[81]
Glucose, sucrose	Potato	Vis-NIR	PLSR	*R*_P_ = 0.90 glucose, *R*_P_ = 0.82 for sucrose	[82]
Defects	Potato	Vis-NIR	LSSVM	90.70%	[83]
Bruise	Potato	Vis-NIR	SVM	100%	[84]
Hardness, resilience, springiness, cohesiveness, gumminess, chewiness	Potato, sweet potato	MIR	LWPLSR	*R*_P_ = 0.80, 0.88, 0.58, 0.57, 0.73 and 0.69 for hardness, resilience, springiness, cohesiveness, gumminess and chewiness	[55]
Moisture content	Potato	Vis-NIR	PLSR	*R*^2^_P_ = 0.98 for moisture content	[85]
Dry matter, starch	Potato, sweet potato	NIR	MLR, PLSR	*R*^2^_P_ = 0.96 for dry matter, *R*_P_**^2^** = 0.96 for starch	[86]
Anthocyanin	Sweet potato	Vis-NIR	MLR	*R*^2^_P_ = 0.87	[87]
Bruise	Potato	Vis-NIR	GLCM	93.75%	[88]
Moisture content, FWC	Sweet potato	Vis-NIR	MLR	*R*^2^_P_ = 0.98 for moisture content, *R*^2^_P_ = 0.93 for FWC	[89]
Cultivar	Sweet potato	NIR	PLSDA	100%	[90]
Moisture content, color	Potato	Vis-NIR	PLSR	*R*^2^_P_ = 0.99 for moisture content, *R*^2^_P_ = 0.99 for colour	[91]
VTC, TCD	Potato, sweet potato	NIR	TBPANN	*R*^2^_P_ = 0.97 for VTC, *R*^2^_P_ = 0.98 for TCD	[92]
Variety	Potato, sweet potato	NIR	PLSDA	≥91.60%	[23]
WBC, SG	Potato, sweet potato	NIR	LWPCR	*R*^2^_P_ = 0.97 for WBC, *R*^2^_P_ = 0.98 for SG	[93]
Moisture content	Potato, sweet potato	NIR	PLSR	*R*^2^_P_ = 0.94	[94]
Blackspot	Potato	Vis-NIR	PLSDA	98.56%	[95]
Starch, glucose, asparagine	Potato	Vis-NIR	PLSR	*R*^2^_P_ = 0.70 for starch, *R*^2^_P_ = 0.51 for glucose, *R*^2^_P_ = 0.70 for asparagine	[96]
Leaf counts, glucose, sucrose, soluble solids, specific gravity	Potato	Vis-NIR	PLSR	*R*_P_ = 0.95 for leaf counts, *R*_P_ = 0.95 for glucose, *R*_P_ = 0.55 for soluble solids, *R*_P_ = 0.95 for sucrose, *R*_P_ = 0.61 for specific gravity	[97]
Sugar-end	Potato	NIR	PLSDA	91.70%	[98]
Cooking time	Potato	Vis-NIR	PLSDA	*R*^2^_P_ = 0.96	[99]
Scab	Potato	NIR	SVM	97.10%	[100]
Hollow heart	Potato	NIR	SVM	89.10%	[101]
Moisture, fat content, color properties, maximum force	Taro chip	NIR	PLSR	*R*^2^_P_ = 0.85–0.97	[102]

LWPLSR—locally weighted partial least squares regression; PLSR—partial least square regression; KNN—k-Nearest Neighbors; LSSVM—least squares support vector machine; PLS-SVM—partial least squares support vector machine; GLCM—gray level co-occurrence matrix; SSC—soluble solid content; SVR—support vector regression; PLSDA—partial least square discriminant analysis; VTC—volatility of tuber compositions; TCD—tuber cooking degree; SMTSM—supervised multiple threshold segmentation model; SVM—support vector machines; MLR—multiple linear regression; BPNN—back-propagation neural network; TBPANN—three-layer back propagation artificial neural network; TA—Total anthocyanin; FWC—freezable water content; *R*_P_—correlation coefficient for prediction; *R*^2^_P_—coefficient of determination for prediction.
